# NKLZ 2.0: Die Weiterentwicklung des Nationalen Kompetenzbasierten Lernzielkatalogs Zahnmedizin als Basis für die Ausgestaltung der neuen Approbationsordnung

**DOI:** 10.1007/s00103-023-03794-1

**Published:** 2023-11-14

**Authors:** Andreas Söhnel, Roland Frankenberger, Lars Kandsperger, Frank Wissing

**Affiliations:** 1 Poliklinik für zahnärztliche Prothetik, Alterszahnheilkunde und medizinische Werkstoffkunde, Universitätsmedizin Greifswald, Walther-Rathenau-Straße 42a, 17475 Greifswald, Deutschland; 2 Medizinischer Fakultätentag der Bundesrepublik Deutschland e. V., Berlin, Deutschland; 3 Poliklinik für Zahnerhaltung, UniversitätsZahnMedizin Marburg, Philipps-Universität Marburg und Universitätsklinikum Gießen und Marburg (UKGM) Standort Marburg, Marburg, Deutschland

**Keywords:** Nationaler Kompetenzbasierter Lernzielkatalog Zahnmedizin, NKLZ, ZApprO, Zahnmedizinische Ausbildung, Kompetenzbasiert, National competence-based learning objectives catalogue for dentistry, NKLZ, ZApprO, Dental education, Competence-based

## Abstract

Der Nationale Kompetenzbasierte Lernzielkatalog Zahnmedizin (NKLZ) wurde 2015 verabschiedet und definiert die Lernziele für die zahnmedizinische Ausbildung in Deutschland. Er legt fest, welche Kompetenzen Studierende erwerben sollen, und dient als Grundlage für die curriculare Gestaltung des Studiums, der Prüfungen und der Unterrichtsmaterialien. Der NKLZ fördert eine umfassende Ausbildung, die neben fachlichem Wissen auch klinische Fähigkeiten und Fertigkeiten, professionelles Verhalten und kommunikative Kompetenzen einschließt. Er trägt zur Vorbereitung angehender Zahnmediziner:innen auf ihren Beruf bei und standardisiert die Ausbildung, um Qualität und Vergleichbarkeit sicherzustellen.

Der vorliegende Artikel beschreibt Hintergründe, Geschichte, Aufbau und die Weiterentwicklung des NKLZ. Derzeit wird der NKLZ zur Version 2.0 in einem mehrstufigen Prozess weiterentwickelt. Seine Struktur orientiert sich am NKLM 2.0, dem Nationalen Kompetenzbasierten Lernzielkatalog Medizin. Ziel ist es, die Anforderungen für die Zahnärztliche Approbationsordnung umfassend abzubilden.

Eine wichtige Voraussetzung für die offizielle Anerkennung des NKLZ als grundlegender Leitfaden für die Ausbildung von Zahnmediziner:innen ist die Verankerung in einer zeitnah zu reformierenden Approbationsordnung Zahnmedizin. Dadurch werden Klarheit und Verbindlichkeit für Lehrende und Studierende geschaffen. Eine solche Verankerung ermöglicht zudem eine bessere Abstimmung zwischen Ausbildungszielen und den Anforderungen der Berufspraxis. Da die Approbationsordnung weniger häufig angepasst wird, bietet die Einbindung des NKLZ die Möglichkeit, Aktualisierungen und Anpassungen des Lernzielkatalogs strukturiert und reguliert vorzunehmen. Dies gewährleistet, dass die Ausbildung den aktuellen Standards und Entwicklungen entspricht.

## Abstract (en)

The National Competence-Based Catalogue of Learning Objectives in Dentistry (NKLZ) was adopted in 2015 and defines the learning objectives for dental training in Germany. It specifies which competences students should acquire and serves as a basis for the curricular design of the study programme, examinations and teaching materials. The NKLZ promotes a comprehensive education that includes clinical skills and abilities, professional behaviour and communication skills in addition to specialist knowledge. It contributes to the preparation of future dentists for their profession and standardizes training to ensure quality and comparability.

This article describes the background, history, structure and further development of the NKLZ. Currently, the NKLZ is being further developed to version 2.0 in a multi-stage process. Its structure is based on the NKLM 2.0, the National Competence-Based Learning Objectives Catalogue for Medicine. The aim is to comprehensively map the requirements for the dental licensing regulations.

An important prerequisite for the official recognition of the NKLZ as a basic guideline for the training of dentists is that it be anchored in a dental licensing regulation that is to be reformed soon. This creates clarity and liability for teachers and students. Such an anchoring also enables better coordination between training objectives and the requirements of professional practice. Since the licensing regulations are modified less frequently, the integration of the NKLZ offers the possibility of updating and adapting the catalogue of learning objectives in a structured and regulated manner. This ensures that training is in line with current standards and developments.

## Einleitung

Der Nationale Kompetenzbasierte Lernzielkatalog Zahnmedizin (NKLZ) ist ein Referenzdokument, welches die Lernziele für die zahnmedizinische Ausbildung in Deutschland definiert. Er wurde zeitgleich zum Nationalen Kompetenzbasierten Lernzielkatalog der Medizin (NKLM) entwickelt und 2015 offiziell verabschiedet, um den Ausbildungsinstitutionen eine klare Struktur und Orientierung für die curriculare Gestaltung des Zahnmedizinstudiums zu geben. Im NKLZ wird beschrieben, welche Kompetenzen Studierende im Verlauf ihres Studiums erwerben sollten und er dient ebenso als Grundlage für die Planung von Lehrveranstaltungen, universitären Prüfungen und die Entwicklung von Unterrichtsmaterialien. Durch seine Kompetenzorientierung fördert der NKLZ eine umfassende Ausbildung, die nicht nur fachliches Wissen, sondern auch klinische Fähigkeiten und Fertigkeiten, professionelles Verhalten und kommunikative Kompetenzen einschließt. Damit trägt der NKLZ fundamental zur Vorbereitung angehender Zahnmediziner:innen auf ihren zukünftigen Beruf bei [1–5].

Die Bedeutung des NKLZ für die zahnmedizinische Ausbildung liegt in seiner Standardisierung und Qualitätssicherung. Durch die stringente Definition klarer Lernziele ermöglicht er eine einheitlichere Ausbildung von Zahnmedizinstudierenden an verschiedenen Hochschulen in Deutschland, ohne individuelle Schwerpunktsetzungen kategorisch zu unterbinden. Der NKLZ gibt bestimmte Kompetenzen vor, die im Studium abgedeckt werden sollten, sodass Studierende über ein angemessenes Kompetenzniveau verfügen, um die Anforderungen des zahnärztlichen Berufs meistern und auch weiterentwickeln zu können. Er bietet den Ausbildungsinstitutionen auch eine Grundlage für die regelmäßige Überprüfung und Aktualisierung ihrer Curricula, um aktuellen Entwicklungen in der Zahnmedizin Rechnung zu tragen.

Der NKLZ spielt eine wichtige Rolle bei der Förderung von Transparenz und Vergleichbarkeit in der zahnmedizinischen Ausbildung. Durch die klare Definition von Lernzielen und Kompetenzen ermöglicht er Studierenden, Lehrenden und potenziellen Arbeitgebern, ein gemeinsames Verständnis der erwartbaren Fähigkeiten und Kenntnisse zu entwickeln. Der NKLZ dient damit der Schaffung einer einheitlichen Ausbildungsqualität. Darüber hinaus bietet er eine Grundlage für die Gestaltung von Prüfungen und Bewertungsmethoden, um Abschlüsse aussagekräftig und vergleichbar zu machen und somit die Qualität der zahnmedizinischen Ausbildung insgesamt zu verbessern.

Der Bedarf zur Überarbeitung der ersten Version des NKLZ von 2015 ergibt sich aus mehreren Gründen: Zum einen liegt der NKLM bereits seit 2021 in einer überarbeiteten und aktualisierten Version vor, sodass eine Weiterentwicklung des NKLZ zur abgestimmten Ausbildung von Human- und Zahnmediziner:innen logische Konsequenz war. Außerdem müssen Lernziele und Kompetenzen in bestimmten Abständen zum einen an die sich wandelnden Anforderungen bzw. technischen Entwicklungen in der Zahnmedizin angepasst werden und zum anderen hinsichtlich ihrer inhaltlichen Relevanz überprüft werden, damit das Studium studierbar bleibt [6].

## Entstehungsgeschichte der Lernzielkataloge

Mit Blick auf den Qualifikationsrahmen für Deutsche Hochschulabschlüsse und den Europäischen Qualifikationsrahmen für lebenslanges Lernen wurde angeregt, auch einen Fachqualifikationsrahmen für das Medizinstudium zu entwickeln. Daraufhin beschlossen der Medizinische Fakultätentag (MFT) und die Gesellschaft für Medizinische Ausbildung (GMA) gemeinsam die Entwicklung eines „Nationalen Kompetenzbasierten Lernzielkatalogs Medizin“ (NKLM) zur Beschreibung eines Kerncurriculums für das Medizinstudium [7]. Entsprechend dieser Empfehlung entschieden auch die Vertreter:innen der Zahnmedizin, ein ähnliches Verfahren durchzuführen, insbesondere im Hinblick auf die Neugestaltung des Unterrichts im Rahmen der bevorstehenden Novellierung der Approbationsordnung und der engeren Verzahnung mit dem medizinischen Studiengang. Somit wurden die Organisationsstrukturen des Arbeitsprozesses für den NKLM auf die Entwicklung des NKLZ übertragen.

Es wurde eine gemeinsame Lenkungsgruppe gebildet, bestehend aus Vertreter:innen des MFT, der Vereinigung der Hochschullehrer für Zahn‑, Mund- und Kieferheilkunde (VHZMK), der Deutschen Gesellschaft für Zahn‑, Mund- und Kieferheilkunde (DGZMK), der Gesellschaft für Medizinische Ausbildung (GMA) sowie des Arbeitskreises für die Weiterentwicklung der Lehre in der Zahnmedizin (AKWLZ). Diese Gruppe beriet und traf im Einvernehmen mit Vertreter:innen von AWMF^1^, BZÄK^2^, BMBF^3^, BMG^4^, BDZM^5^, HRK^6^, KMK^7^, GMK^8^ sowie des VUD^9^ Entscheidungen, während die eigentliche Entwicklungsarbeit in interdisziplinären Arbeitsgruppen stattfand. Deren Entwürfe wurden mit der Lenkungsgruppe diskutiert und einem Konsensusprozess unterzogen. Nach Erstellung eines Gesamtentwurfs erfolgte ein NKLZ-Konsensusverfahren (10/2014 bis 04/2015). Der endgültige NKLZ 1.0 wurde auf dem ordentlichen Medizinischen Fakultätentag 2015 in Kiel verabschiedet und den medizinischen Fakultäten zur Verfügung gestellt [8].

Im Rahmen des „Masterplans Medizinstudium 2020“ wurde der Auftrag zur Weiterentwicklung des NKLM formuliert [9]. Der Medizinische Fakultätentag (MFT) und das Institut für medizinische und pharmazeutische Prüfungsfragen (IMPP) wurden beauftragt, den NKLM und die Gegenstandskataloge (GK) weiterzuentwickeln. Unter Beteiligung verschiedener Akteure, Arbeitsgruppen, Experten sowie anhand internationaler Empfehlungen zur Curriculumsentwicklung wurde der NKLM zum NKLM 2.0 weiterentwickelt und im Jahr 2021 veröffentlicht.

Der NKLM 2.0 zielt darauf ab, diejenigen Kompetenzen zu beschreiben, die von allen Medizinstudierenden erworben werden sollen. Er stellt kein lineares Katalogsystem mehr dar, sondern enthält kompetenzbasierte Querverbindungen zwischen seinen Lernzielen und bietet umfangreiche Filter- und Suchfunktionen. Der NKLM soll in der zukünftigen reformierten Approbationsordnung für Ärztinnen und Ärzte für den Kernbereich des Medizinstudiums rechtlich verbindlich werden, was im aktuellen Referentenentwurf auch bereits festgeschrieben ist. Bereits jetzt werden die medizinischen Fakultäten ermutigt, den Katalog als Grundlage für ihre curriculare Weiterentwicklung zu nutzen. Neue Elemente und Inhalte sollten erprobt werden und die Erfahrungen systematisch in die Weiterentwicklung des NKLM einfließen. Die Fakultäten werden auch dazu eingeladen, ihre eigenen Lernzielkataloge mit dem NKLM abzugleichen („Mapping“) und zur kontinuierlichen Weiterentwicklung beizutragen.

Der NKLM wird elektronisch bereitgestellt, um eine Verknüpfung mit fakultären Lernzielkatalogen und den Gegenstandskatalogen des IMPP zu ermöglichen. Fachgesellschaften und interessierte Dritte können ebenfalls ihre eigenen Lernzielkataloge mit dem NKLM abgleichen. Die inhaltliche Weiterentwicklung des NKLM erfolgt durch den MFT auf einer technischen Plattform in Kooperation mit dem Learning Opportunities, Objectives and Outcome Platform(LOOOP)-Projekt der Charité und in Abstimmung mit der GMA und weiteren relevanten Akteuren und Ministerien auf Bundes- und Landesebene sowie dem IMPP, um eine Kongruenz mit dem Gegenstandskatalog sicherzustellen.

Auf dem Weg zum NKLM 2.0 wurde die gesamte Kapitelstruktur überarbeitet und von ehemals 21 Kapiteln auf 8 reduziert. Die Gliederungsebenen Kompetenzen, Teilkompetenzen und, je nach Kapitel, Konsultationsanlässe, Erkrankungen und Lernziele wurden aus der alten Struktur in die neue überführt und jeweils mit präzisierenden Detailinformationen und Erläuterungen versehen. Deskriptoren geben in den Kapiteln zu Konsultationsanlässen und Erkrankung an, welche Aspekte Studierende im Verlauf ihres Medizinstudiums im Kerncurriculum auf Wissensebene oder mit Handlungskompetenzen beherrschen sollen. Zudem werden für übergeordnete krankheitsbezogene Lernziele sowie übergeordnete Kompetenzen Zeitpunkte im Studium definiert, zu denen diese in einer definierten Kompetenztiefe erworben sein sollen.

Aktuell wird der NKLM hin zu einer Version 2.1 (Veröffentlichung in 2024) und späteren Version 3.0 weiterentwickelt, um Aktualisierungen vorzunehmen, inhaltliche Redundanzen zu vermeiden und eine Fokussierung auf ein studierbares Kerncurriculum umzusetzen.

NKLZ und NKLM sind in ihrer jeweils aktuellen Fassung im Internet frei abrufbar (www.nklz.de bzw. www.nklm.de).

## Aufbau NKLZ 1.0

Der NKLZ besteht in seiner ersten und derzeit gültigen Version aus 26 Kapiteln, von denen die ersten 4 eine allgemeine Einführung, eine Erläuterung von Kompetenzen, Rollen und Lernzielen, eine Beschreibung der Prüfungsmethoden sowie der Qualitätsanforderungen für Institutionen der zahnärztlichen Ausbildung abbilden. Die nachfolgenden Kapitel listen die eigentlichen Kompetenzen, Teilkompetenzen sowie Lernziele/Konsultationsanlässe/Erkrankungen auf. Insgesamt werden knapp 160 Kompetenzen, 268 Teilkompetenzen, 1785 Lernziele, 71 Konsultationsanlässe und 131 Erkrankungen mit zahnmedizinischem Bezug aufgeführt. Die Kapitel 5 bis 11 beschreiben die 7 verschiedenen beruflichen Rollen von Zahnärzt:innen und basieren auf dem kanadischen Rahmenkonzept „Canadian Medical Education Directives for Specialists“ (CanMEDS; [10]). Ursprünglich für fachärztliche Kompetenzen entwickelt, wurde das Modell auf die Zahnmedizin übertragen und im Kontext des Entwurfs der neuen Approbationsordnung für Zahnärzte sowie der zahnärztlichen Muster-Berufsordnung an das Kompetenzniveau von Absolvent:innen der zahnmedizinischen Ausbildung angepasst und weiterentwickelt.

Eine zentrale Rolle im NKLZ nimmt der „ZahnMedizinische Experte“ ein, der sowohl auf zahnärztliches als auch medizinisches Wissen, klinische Fähigkeiten und Fertigkeiten sowie auf professionelle Haltungen zurückgreift. Zusätzlich zu dieser Rolle gibt es weitere Rollen wie „Gelehrter“, „Kommunikator“, „Mitglied eines Teams“, „Gesundheitsberater und -fürsprecher“, „Verantwortungsträger und Manager“ sowie „Professionell Handelnder“. Diese Rollen dienen der bestmöglichen Umsetzung einer patientenzentrierten Gesundheitsversorgung. Im NKLZ werden die übergeordneten Kompetenzen, Teilkompetenzen und Lernziele für diese 7 Zahnarztrollen bis zum Abschluss des Studiums beschrieben. Sie befähigen zur zahnärztlichen Weiterqualifikation und sollen im Sinne des „lebenslangen Lernens“ und der Reflexion der eigenen Kompetenzen kontinuierlich weiterentwickelt werden. Die Rolle des „ZahnMedizinischen Experten“ ist im Abschnitt II des NKLZ (Kapitel 12 bis 22) mit den zentralen Themenbereichen des relevanten Wissens, des wissenschaftlichen Erkenntnisgewinns, der klinischen Fähigkeiten und Fertigkeiten in den Bereichen der Prävention, Diagnose, Behandlungsplanung und Therapie sowie der (zahn-)ärztlichen Grundhaltungen abgebildet. Diese Fähigkeiten und Fertigkeiten sollen während der Ausbildung erworben und gefestigt werden.

Die Kapitel 23a bis 26 bilden den Abschnitt III und behandeln wichtige Anlässe der zahnärztlichen Konsultation sowie die präventive, diagnostische und therapeutische Versorgung relevanter Erkrankungen. In der zahnärztlichen Ausbildung wird bereits während des Studiums besonderer Wert auf die aktive Behandlung dieser Erkrankungen gelegt. In den klinischen Semestern sowie in den Staatsexamensprüfungen führen Studierende unter Anleitung/Aufsicht eigenständig präventive, diagnostische und invasiv therapeutische Maßnahmen an Patient:innen durch, was in diesem Abschnitt des Lernzielkatalogs unter Berücksichtigung der angestrebten Kompetenzebenen detailliert beschrieben wird.

Die Abgrenzung zu Abschnitt II erfolgt primär krankheitsspezifisch: Während in Abschnitt II grundlegende Prinzipien und fundamentale Fähigkeiten und Fertigkeiten behandelt werden, behandelt Abschnitt III krankheitsspezifische Kenntnisse und Fertigkeiten in den Bereichen Prävention, Diagnostik und Therapie relevanter Erkrankungen, einschließlich allgemeinmedizinischer Aspekte, die von kompetenten und fortbildungsfähigen Absolvent:innen erwartet werden. Bei der Auswahl der Erkrankungen für den Abschnitt III wurden Kriterien wie Häufigkeit, potenzielle Letalität, nachhaltige Einschränkung der Lebensqualität sowie zahnärztlicher und auch ärztlicher Handlungsbezug berücksichtigt. Zudem wurden seltene Erkrankungen in die Lernziele aufgenommen, wobei in der Vermittlung der Kompetenzen im Umgang mit seltenen Erkrankungen der Fokus auf dem methodischen Zugang zu spezifischen Informationsquellen und -techniken liegt.

Der „Masterplan Medizinstudium 2020“ und der Referentenentwurf der Approbationsordnung sahen eine klare Definition der Lernziele vor, die in den verschiedenen Studienabschnitten, spätestens im Rahmen des Kerncurriculums, vermittelt werden sollen, sodass bereits 2018 mit der Weiterentwicklung des NKLM begonnen wurde [9, 11]. Auch sahen beide Dokumente vor, dass mit dem Inkrafttreten der neuen Ärztlichen Approbationsordnung (ÄApprO) der NKLM bundesweit ein Kerncurriculum für die medizinische Ausbildung festlegt wird. Somit bestand das Ziel der Weiterentwicklung darin, den Inhalt dieses Kerncurriculums präzise zu definieren. Einerseits sollten alle erforderlichen Kompetenzen von allen Studierenden erworben werden, andererseits sollte ausreichend Spielraum für fakultäre und persönliche Schwerpunkte außerhalb des verbindlichen Kerncurriculums bleiben. In einem 6‑stufigen Prozess wurde der NKLM von 2018 bis zur endgültigen Verabschiedung im März 2021 unter diesen Maßgaben überarbeitet.

In der Zahnmedizin wurde Anfang 2019 ein Referentenentwurf zur Reform der zahnärztlichen Approbationsordnung (ZApprO) vorgelegt, der bereits im Juni des Jahres vom Bundesrat verabschiedet und ratifiziert wurde [12]. Im Gegensatz zur Medizin wurde weder in diesem Entwurf noch im endgültigen Gesetzestext der NKLZ erwähnt oder als konsentiertes Kerncurriculum aufgeführt. Aufgrund der recht kurzfristigen Umsetzung der neuen ZApprO (Start Oktober 2021) wurde eine Weiterentwicklung des NKLZ im Jahr 2019 nicht gezielt verfolgt. 2020/2021 herrschte aufgrund der Coronapandemie eine angespannte Situation an den universitätszahnmedizinischen Standorten, in der einer Weiterentwicklung des NKLZ keine Priorität zugeordnet werden konnte. Die ursprünglich geplante NKLZ-Weiterentwicklung wurde jedoch nie aus den Augen verloren und auch adressiert. Im MFT-Positionspapier „Zukunft des Zahnmedizinstudiums“ wurde explizit gefordert, dass der NKLZ ein zentrales Element für die inhaltliche Gestaltung des Zahnmedizinstudiums sei. Um dieser Aufgabe gerecht zu werden, muss der Katalog regelmäßig restrukturiert und aktualisiert werden [13]. Dadurch kann der NKLZ das zahnmedizinische Studium normativ begleiten und sich dabei dynamisch an die inhaltliche Weiterentwicklung anpassen. Die Zahnärztliche Approbationsordnung sollte diesem fortschrittsdienlichen Element Raum geben und somit lediglich den äußeren Rahmen für eine zuverlässige Umsetzung von Ausbildung und Prüfungen bilden. Auch wurden eine direkte Anbindung an die ZApprO sowie eine ausreichende und gesicherte Finanzierung der Weiterentwicklung gefordert.

Während im Rahmen der alten Approbationsordnung die zahnärztlichen Prüfungen mündlich und praktisch bestritten wurden, enthält mit der neuen ZApprO der dritte Abschnitt der zahnärztlichen Prüfung nun auch einen schriftlichen Teil, der wie in der Medizin durch das IMPP organisiert wird. Hierfür wird derzeit vonseiten des IMPP an der Erstellung eines Gegenstandkatalogs Zahnmedizin (GK ZM) gearbeitet, der auf dem NKLZ basiert. Da die schriftliche Prüfung mit 200 Fragen die sogenannten „Außer-Haus-Fächer“ wie innere Medizin, Dermatologie, Pathologie und die neu etablierten Querschnittsbereiche (QB) abdeckt, wird für die Erstellung der Gegenstände auch auf Anteile des NKLM 2.0 zurückgegriffen. Somit ist es auch hier im Sinne eines „constructive alignment“ wichtig (Abstimmung von Lernzielen, Lehr‑/Lernaktivitäten und Assessment aufeinander), dass der NKLZ 2.0 Anteile des NKLM 2.0 übernimmt, um eine rechtssichere und umfassende schriftliche Prüfung zu gewährleisten.

## Weiterentwicklung zum NKLZ 2.0

Ende 2021 begann unter der Federführung des MFT die Weiterentwicklung des NKLZ 1.0 zur 2.0-Version. Im ersten Schritt wurde eine Redaktionsgruppe mit mandatierten Vertreter:innen der verschiedensten Akteursgruppen gebildet. Hierzu gehören die Deutsche Gesellschaft für Zahn‑, Mund- und Kieferheilkunde (DGZMK), die Bundeszahnärztekammer (BZÄK), die Vereinigung der Hochschullehrer für Zahn‑, Mund- und Kieferheilkunde (VHZMK), das IMPP, die großen zahnmedizinischen Fachgruppierungen sowie studentische Vertreter:innen des Bundesverbands der Zahnmedizinstudierenden (BdZM).

In den ersten Sitzungen wurden das allgemeine Vorgehen, die Entscheidung zur prinzipiellen Übernahme der neuen Kapitelstruktur des NKLM sowie die Besetzung der 23 Arbeitsgruppen erarbeitet. Jede Arbeitsgruppe besteht aus mehreren Mitgliedern, einer/m Arbeitsgruppenleiter:in sowie einem „Paten“ oder einer „Patin“. Die Aufgabe der Arbeitsgruppenleitenden ist es, als Ansprechperson für das jeweilige Kapitel der Redaktionsgruppe zur Verfügung zu stehen, den Arbeitsprozess der Mitglieder zu überblicken sowie den Fortschritt zu überwachen. Die Paten sind u. a. Teil der Redaktionsgruppe und haben die Aufgabe, bei strittigen Fragen innerhalb der Arbeitsgruppe bzw. zwischen Redaktionsgruppe und Arbeitsgruppe zu vermitteln. Insgesamt wurden mehr als 130 Kolleg:innen angesprochen, bei der Überarbeitung mitzuarbeiten. Unterstützend dazu findet über die Gremien des MFT, insbesondere den Ausschuss Lehre, und die Geschäftsstelle ein regelmäßiger Austausch zwischen den Prozessen der NKLZ- und der NKLM-Weiterentwicklung statt.

Die Weiterentwicklung erfolgt in einem 4‑stufigen Prozess: In Phase 1 werden den Arbeitsgruppen die Kapitel des NKLZ nach alter Struktur präsentiert, um über die bereits vorhandenen Struktureinheiten wie Kompetenzen, Teilkompetenzen und Lernziele zu entscheiden. Es wird entschieden, ob die Struktureinheiten a) unbearbeitet für den NKLZ 2.0 übernommen werden, b) inhaltlich oder semantisch bearbeitet werden, c) gegebenenfalls in mehrere Struktureinheiten aufgeteilt werden oder d) gelöscht werden. Die Bearbeitung wird durch jedes Mitglied vorgenommen, sodass möglicherweise unterschiedliche Verfahrensempfehlungen pro Struktureinheit vorliegen. Diese Unterschiede sollen in einer finalen Online-Abschlusssitzung durch die Gruppe unter Leitung des Arbeitsgruppenleiters oder der Arbeitsgruppenleiterin konsentiert werden, um ein gemeinsames Ergebnis der Bearbeitung der Phase 1 zu erreichen.

In Phase 2 können die im ersten Schritt zur Bearbeitung bzw. zur Aufteilung vorgesehenen Struktureinheiten direkt bearbeitet werden. Phase 1 und 2 orientieren sich dabei an der alten Struktur des Lernzielkataloges. Erst in Phase 3 werden die bereits bearbeiteten Struktureinheiten in der neuen Kapitelstruktur dargestellt und, ähnlich wie in Phase 1, im neuen Kontext auf Doppelungen, mögliche Präzisierungen, Aufteilungen oder Löschung aufgrund von jetzt erst sichtbaren Redundanzen erneut von allen Arbeitsgruppenmitgliedern bewertet.

**Figure 1:**
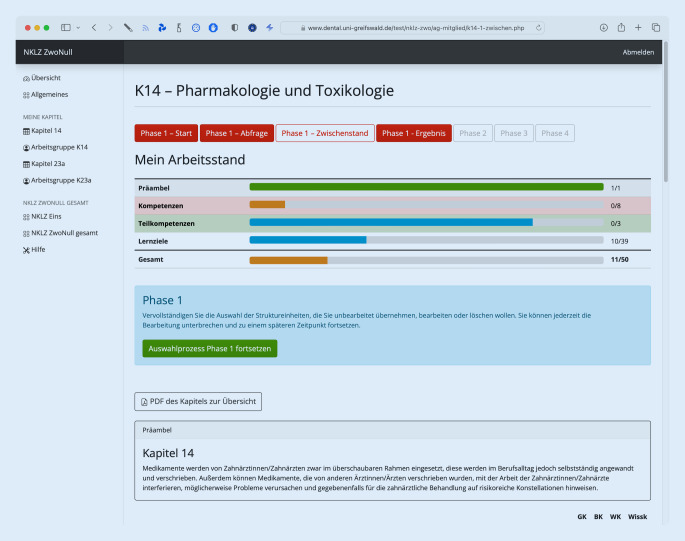

Um eine moderne und zeitgemäße Weiterentwicklung zu etablieren und auf einen aufwändigen Austausch und schwierigen Abgleich von Excel-Tabellen zu verzichten, wurde eine auf den aktuellen Webtechniken HTML, PHP und MySQL beruhende, webbasierte Reviewplattform beauftragt und in Kooperation mit der Universität Greifswald erstellt. Jedes Arbeitsgruppenmitglied hat für das Reviewtool einen passwortgeschützten Zugang auf die individuell freigeschalteten Funktionen und wird hier anhand einer durchdachten Prozesslogik durch die einzelnen Arbeitsschritte geleitet (Abb. 1, 2 und 3). Zur Schulung der Arbeitsgruppenmitglieder und der Arbeitsgruppenleiter:innen wurden mehrere Bildschirmaufnahmen gemacht, die allen Mitgliedern auf einer Hilfeseite zur Verfügung stehen. Darüber hinaus ist ein Support via E‑Mail ständig verfügbar und konnte in der Regel die meisten Probleme zeitnah lösen.

**Figure 2:**
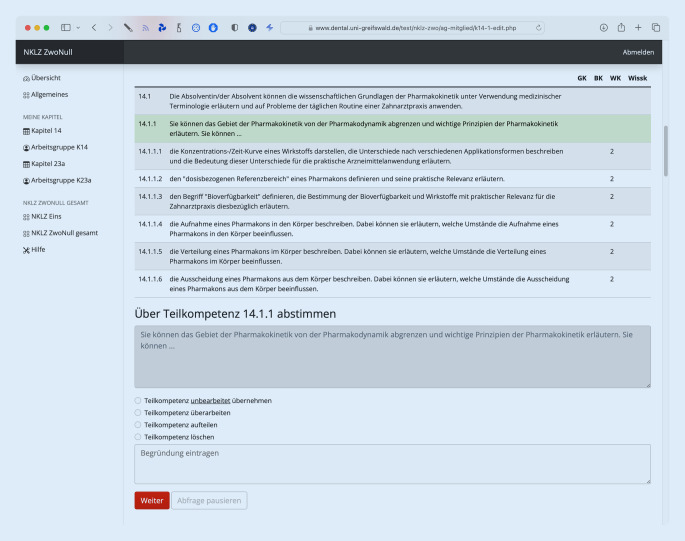

**Figure 3:**
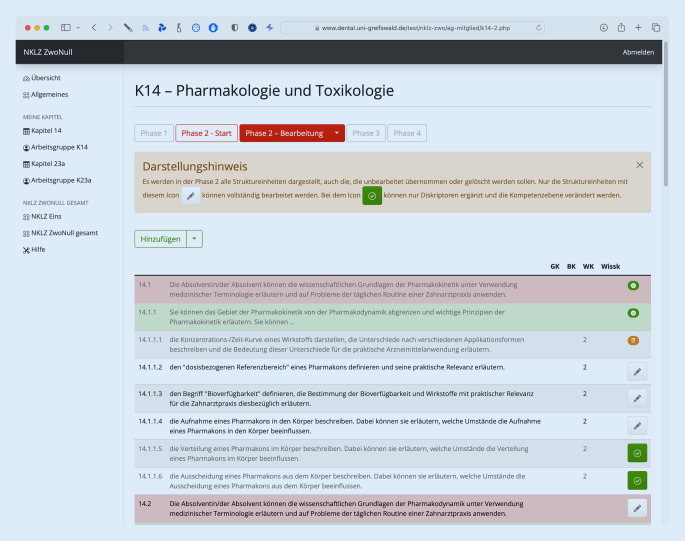

Die Weiterentwicklung des NKLZ befindet sich – Stand Juni 2023 – in Phase 2 und wird insgesamt voraussichtlich bis zum Ende des Jahres 2023 durch die Arbeitsgruppen abgeschlossen werden, bevor der Katalog in der Version 2.0 den Fakultäten und Fachgruppen zur inhaltlichen Sichtung übergeben werden wird.

Phase 1 wurde im Oktober 2022 gestartet und sollte eigentlich bis zum Januar 2023 abgeschlossen sein. Bei einer Arbeitsstandanalyse Mitte Dezember 2022 zeigte sich ein stark heterogenes Bild: Einige Arbeitsgruppenmitglieder hatten in kürzester Zeit alle Struktureinheiten bewertet, während die meisten noch nicht angefangen hatten. Nach direkter Ansprache per E‑Mail konnte Ende Februar 2023 die Bearbeitung der Phase 1 durch alle Mitglieder verzeichnet werden. Für die arbeitsgruppeninterne finale Absprache und Einigung auf ein gemeinsames Endergebnis wurden alle Arbeitsgruppenleiter:innen erneut mit den Zusatzfunktionen für Leiter:innen des Reviewtools vertraut gemacht und um eine baldige Beendigung gebeten. Da auch dieser Prozess teilweise schleppend voranging, wurden die jeweiligen Abschlusssitzungen durch den MFT selbst terminlich festgelegt und moderiert, um die Arbeitsgruppenleiter in ihrer Arbeit zu unterstützen. Anfang Mai 2023 hatten alle Arbeitsgruppen ein konsensuales Ergebnis der Phase 1 erstellt. In einer ersten Sichtung der Abstimmungsergebnisse zeigt sich auf alter Kapitelebene ein gemischtes Bild: Während manche Arbeitsgruppen ihr Kapitel fast vollständig und ohne große Änderungen übernehmen wollen, möchte ein Großteil der Arbeitsgruppen (ca. 80 %) den Kapitelinhalt bearbeiten. Sehr selten wurden Struktureinheiten identifiziert, die zur Löschung vorgesehen wurden.

Derzeit werden die letzten Vorbereitungen und Programmierungen für Phase 2 vorgenommen, um zeitnah alle Arbeitsgruppenmitglieder für Phase 2 freizuschalten. Auch wenn die Bearbeitung von Phase 1 länger als geplant gedauert hat, werden die bis dato gesammelten Erfahrungen in die Optimierung des weiteren Prozesses fließen: enges Zeitmanagement mit klarer Terminabsprache und frühzeitiger Unterstützung, erklärende Schulungsangebote für jede neue Phase online als Videokonferenz und deren Aufzeichnung zur Erstellung weiteren Schulungsmaterials.

## Vorteile durch die Orientierung am NKLM und die Nutzung des Onlinetools

Es ist von fundamentaler Bedeutung, dass sich der NKLZ 2.0 am Lernzielkatalog der Humanmedizin orientiert und ein beständiger Abgleich zwischen beiden Katalogen stattfindet. Nur eine enge Verbindung zwischen den beiden Katalogen ermöglicht einen ganzheitlichen Ansatz in der Patientenversorgung. Zahnärztinnen und Zahnärzte arbeiten oft interdisziplinär mit Ärzt:innen zusammen, um eine umfassende Behandlung zu gewährleisten [13]. Eine gemeinsame Grundlage von Lernzielen und Kompetenzen erleichtert die Zusammenarbeit und fördert eine patientenzentrierte Versorgung. Zudem bietet die Orientierung am NKLM die Möglichkeit, bewährte Konzepte und Methoden aus der medizinischen Ausbildung zu übernehmen und spezifisch auf die Bedürfnisse der Zahnmedizin anzupassen sowie die ausgeprägten Erfahrungen zur Entwicklung von praktischen Fertigkeiten in der zahnmedizinischen Ausbildung in medizinische Ausbildungsstrukturen zu übertragen. Durch den Austausch von bewährten Praktiken und deren Anpassung an aktuelle Entwicklungen in der medizinischen Ausbildung können Synergien genutzt und die Qualität der zahnmedizinischen Ausbildung weiter verbessert werden. Schließlich trägt der NKLZ dazu bei, eine einheitliche Ausbildung in der Zahnmedizin sicherzustellen. Einheitliche Lernziele und Kompetenzen ermöglichen Vergleichbarkeit und Transparenz in der Ausbildung von Zahnmediziner:innen in verschiedenen Bildungseinrichtungen und fördern eine hohe Qualität der zahnmedizinischen Versorgung in Deutschland.

Derzeit zeichnet es sich ab, dass beide relevanten Kataloge, der NKLZ und der Gegenstandskatalog Zahnmedizin, mit demselben Onlinetool und somit der gleichen Datenstruktur weiterentwickelt werden. Die Verwendung eines solchen Tools ermöglicht eine effiziente Aktualisierung der Kataloge und spart Zeit und Ressourcen. Durch die einheitliche Struktur und Standards gewährleistet ein Tool die Konsistenz der Kataloge. Ein weiterer Vorteil eines Tools mit einer gemeinsamen Datenbasis ist die Möglichkeit der kontinuierlichen Aktualisierung der Kataloge. Neue Erkenntnisse und Entwicklungen können so in kurzer Zeit integriert und die Lernziele und Inhalte stets auf dem neuesten Stand gehalten werden. Dies ist besonders wichtig, um den aktuellen Anforderungen der Zahnmedizin gerecht zu werden und den Studierenden das aktuelle Fachwissen zu vermitteln. Während für die Weiterentwicklung des NKLZ ein dafür spezifisch adaptiertes Tool verwendet wird, ist der veröffentlichte NKLZ (ebenso wie der NKLM) plattformneutral und kann für die weitere Nutzung und Curricularentwicklung in verschiedenen datenbankbasierten Tools importiert werden (z. B. MyNKL, LOOOP, MERLIN, Excel; [14, 15]).

Die Verwendung eines Tools und einer Datenbasis schafft zudem Transparenz und erleichtert den Zugang zu den Katalogen. Lehrende, Studierende und andere Beteiligte können problemlos auf die Lernziele und Inhalte zugreifen. Durch die systematische Evaluierung und Qualitätssicherung der Kataloge können Schwachstellen identifiziert und verbessert werden, um eine kontinuierliche Verbesserung der Ausbildung zu gewährleisten. Insgesamt erleichtert die Verwendung eines Tools und einer Datenbasis die Weiterentwicklung und Verwaltung der Kataloge, ermöglicht eine aktuelle und qualitativ hochwertige Ausbildung und verbessert die Kommunikation und Zusammenarbeit zwischen den verschiedenen Akteuren im Bildungsprozess. Ohne die schnelle Bereitstellung und die stringente Nutzung des neuen Reviewtools wäre die Arbeit trotz leichter Verzögerungen niemals in dieser Zeit zu bewältigen gewesen.

## Fazit

Die Weiterentwicklung des NKLZ zur Version 2.0 ist auf einem guten Weg. Der NKLZ 2.0 wird in einem strukturierten Prozess an der Kapitelstruktur des NKLM 2.0 ausgerichtet und er wird die Erfordernisse eines Kerncurriculums für die neue Zahnärztliche Approbationsordnung umfassend abbilden. Darüber hinaus ist die Verankerung des NKLZ in einer zeitnah zu reformierenden Approbationsordnung Zahnmedizin von großer Bedeutung. Nur eine solche Verankerung stellt sicher, dass der NKLZ als grundlegender Leitfaden für die Ausbildung von Zahnmediziner:innen offiziell anerkannt wird. Dies schafft Klarheit und Verbindlichkeit für alle beteiligten Akteure, einschließlich der Lehrenden und Studierenden. Auch ermöglicht eine solche Verankerung eine bessere und zeitnahe Abstimmung zwischen den Ausbildungszielen und den Anforderungen der Berufspraxis, da die Approbationsordnung eher in größeren Zeitabschnitten angepasst werden wird. Die Verankerung ist auch wichtig, um die inhaltliche und strukturelle Verknüpfung mit dem Medizinstudium politisch zu unterstreichen. Zahnmedizin und Humanmedizin entwickeln sich ständig weiter und es ist wichtig, dass die Ausbildungsinhalte und -ziele den aktuellen Bedürfnissen und Herausforderungen des Berufsstandes Rechnung tragen. Durch die Einbeziehung des NKLZ in die Approbationsordnung können Aktualisierungen und Anpassungen des Lernzielkatalogs in einer strukturierten und regulierten Weise erfolgen, um sicherzustellen, dass die Ausbildung den aktuellen Standards und Entwicklungen entspricht. Darüber hinaus dient eine Verankerung des NKLZ in der Approbationsordnung auch als Qualitätsmaßstab für die Ausbildungsinstitutionen. Es wird sichergestellt, dass die Ausbildungsinhalte und -ziele den nationalen Standards entsprechen und eine hohe Qualität gewährleistet ist. Dies trägt zur weiteren Professionalisierung der zahnmedizinischen Ausbildung bei und stärkt das Vertrauen in den Berufsstand.

## Footnotes

1 Arbeitsgemeinschaft der Wissenschaftlichen Medizinischen Fachgesellschaften e. V.

2 Bundeszahnärztekammer.

3 Bundesministerium für Bildung und Forschung.

4 Bundesministerium für Gesundheit.

5 Bundesverband der Zahnmedizinstudierenden in Deutschland e. V.

6 Hochschulrektorenkonferenz.

7 Kultusministerkonferenz.

8 Gesundheitsministerkonferenz.

9 Verband der Universitätsklinika Deutschlands e. V.
